# Dietary total, plant, and animal protein intake in relation to cardiovascular outcomes and inflammatory factors in elderly men: A cross‐sectional study

**DOI:** 10.1002/fsn3.3837

**Published:** 2023-11-20

**Authors:** Hanieh Abbasi, Noushin Fahimfar, Pamela J. Surkan, Leila Azadbakht

**Affiliations:** ^1^ Department of Community Nutrition, School of Nutritional Sciences and Dietetics Tehran University of Medical Sciences Tehran Iran; ^2^ Osteoporosis Research Center, Endocrinology and Metabolism Clinical Sciences Institute Tehran University of Medical Sciences Tehran Iran; ^3^ Endocrinology and Metabolism Research Center, Endocrinology and Metabolism Clinical Sciences Institute Tehran University of Medical Sciences Tehran Iran; ^4^ Department of International Health John Hopkins School of Public Health Baltimore Maryland USA; ^5^ Diabetes Research Center, Endocrinology and Metabolism Clinical Sciences Institute Tehran University of Medical Sciences Tehran Iran; ^6^ Department of Community Nutrition, School of Nutrition and Food Science Isfahan University of Medical Sciences Isfahan Iran

**Keywords:** cardiovascular risk factors, elderly, inflammation, protein intake

## Abstract

The source and amount of protein intake may influence cardiovascular and inflammatory risk, especially in elders who are often more vulnerable. However, findings on elders have been contradictory. Therefore, we examined the association between dietary total, plant, and animal protein intake in relation to cardiovascular outcomes and inflammatory factors in elderly men. The present cross‐sectional study included 357 elderly men. A validated and reliable food frequency questionnaire (FFQ) was used to assess dietary intake. All biochemical factors including triglycerides (TG), fasting blood sugar (FBS), high‐sensitivity C‐reactive protein (hs‐CRP), interleukin 6 (IL6), and tumor necrosis factor‐α (TNF‐α) were measured. Waist circumference (WC) and blood pressure (BP) were also assessed. A significant inverse association was found between animal protein intake and systolic blood pressure (SBP; OR: 0.62; 95% CI: 0.42, 0.91; *p*trend = .014). There were significant inverse associations between plant protein intake and WC (OR: 0.34; 95% CI: 0.17, 0.68; *p*trend < .001), FBS (OR: 0.51; 95% CI: 0.29, 0.89; *p*trend = .018) and Hs‐CRP (OR: 0.39; 95% CI: 0.21, 0.70; *p*trend = .002). Moreover, significant inverse associations were also found between total protein intake and SBP (OR: 0.54; 95% CI: 0.33, 0.86; *p*trend = .010) and total protein and Hs‐CRP (OR: 0.50; 95% CI: 0.28, 0.88; *p*trend = .015). In elderly men, a high dietary intake of plant protein was associated with lower odds of having high WC, FBS, and Hs‐CRP. In addition, high dietary intake of animal protein was associated with higher odds of having a high SBP level, which was explained by higher intake of dairy products.

## Graphical abstract

Plant protein may be more beneficial for cardiovascular health as it has made more beneficial changes in cardiovascular risk factors.

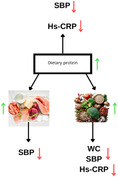

## 1 INTRODUCTION

Population aging in Iran is accelerating, which is illustrated by the fact that the proportion of people aged ≥60 exceeded 10% of the total population in 2022 (Doshmangir et al., 2023). This trend results in multiple challenges for the population as well as concerns about the pace of future economic growth, the sustainability and financial integrity of healthcare, and the well‐being of the elderly (Noroozian, 2012). Based on reports from the American Heart Association, cardiovascular diseases (CVDs) are a global problem, especially in older populations (Mozaffarian et al., 2015). Worldwide, the number of people with CVDs has increased from 271 million in 1990 to 523 million in 2019 (Roth et al., 2020). Approximately two‐thirds of CVD‐related deaths occur in the elderly (American Heart Association, 2013). Estimates predict that CVD will be the cause of more than 23 million (about 30.5%) deaths by 2030 worldwide (Lozano et al., 2012; Mozaffarian et al., 2015). According to The Global Burden of Disease (GBD's) previous reports in 2010 and 2015, CVD is responsible for 20%–23% of the burden of diseases in Iran (Naghavi et al., 2014; Namazi Shabestari et al., 2015; Shams‐Beyranvand et al., 2017). Moreover, based on national statistics in 2015, the prevalence of CVDs among elderly from Iran was 39.9% (Shamsi et al., 2017).

The increased prevalence of CVDs is thought to be related to socioeconomic and cultural changes, nutrition, insufficient physical activity, and increased metabolic and physical risk factors (Sarrafzadegan & Mohammadifard, 2019). Therefore, much interest exists in the role of nutrition in prevention and treatment of CVDs (Sala‐Vila, 2015; Willet, 1999). According to Tappia et al., nutritional factors are responsible for approximately 40% of all cases of CVDs (Willet, 1999). The source and amount of protein in one's diet could affect cardiovascular risk factors (Chesney, 2015; Naghshi, 2020).

According to a review, CVD risk could be decreased by dietary patterns that provide more plant protein sources (including unprocessed animal proteins), compared with the typical American diet (Chesney, 2015). Another study suggested that both processed and red meat were associated with increased CVD risk (Riccardi et al., 2022). In addition, a cohort study of 29,682 participants revealed that consuming plant proteins and fish were associated with lower risk of CVD incidence (Zhong et al., 2021). A recent meta‐analysis on the effect of animal and plant proteins on CVD risk factors showed that consumption of plant proteins may have protective effects against CVD risk factors (Lamberg‐Allardt et al., 2023). However, it has been suggested that some groups of animal proteins like poultry and dairy products may be more effective in controlling CVD risk factors than some plant proteins like nuts (Zhubi‐Bakija et al., 2021).

Thus, the association between source and amount of protein intake and CVD risk factors are still unclear. In addition, few studies have been conducted among elderly. The aforementioned reasons justify evaluating the association between the amount and sources of protein intake and cardiovascular outcomes and inflammatory factors in an elderly population. In addition, cardiovascular disease predominantly affects men; that is, for most age groups, rates of cardiovascular disease in men are higher than in women (Albrektsen et al., 2016). Therefore, the objective of this study was to examine the cross‐sectional association between the amount and sources of protein intake and cardiovascular risk factors in elderly men.

## 2 MATERIALS AND METHODS

### 2.1 Study population

In this cross‐sectional study, 365 men were included from health centers in southern Tehran, Iran (March to August 2017). Ethics approval for the study was obtained from Tehran University of Medical Sciences (TUMS), as health centers in south of Tehran are supervised by TUMS (grant number: 48040). Men who were referred to health centers for primary healthcare were contacted by staff for inclusion in the present study. All participants were asked to provide written informed consent to be included in the study. Inclusion criteria were being: (a) an Iranian male; (b) over 60 years old; (c) no self‐reported previous diagnosis of malignant diseases (e.g., cancer); and (d) no change in usual diet as a result of disease or per dietitian recommendation.

Hypertension was the main dependent variable used to calculate study sample size (as the highest sample size would be obtained using this variable) (Mehrabani et al., 2017). A total sample size of 340 participants was estimated. Clustered random sampling was used to determine the number of participants selected from each health center. After data collection (*n* = 365), participants who had very low calorie intake (<800 kcal/day) or high calorie intake (>4200 kcal/day) were excluded and 357 participants remained for statistical analysis.

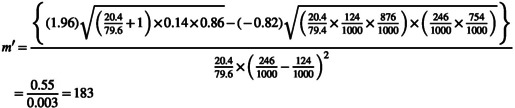




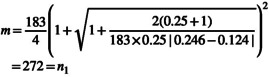

$$\begin{array}{c} m\times r=n_{2}\\ \;=68 \end{array}$$

$$272+68=340=n_{1}+n_{2}$$

### 2.2 Dietary assessment

All participant nutritional information was obtained using 168‐item semiquantitative food frequency questionnaire (FFQ) through face‐to‐face interviews by a trained nutritionist. Dietary intakes were changed from serving sizes and household measures to grams. A modified version of the NUTRITIONIST IV software (version 7.0; N‐Squared Computing), which was designed for Iranian foods, was used to compute nutrient intake (Abshirini et al., 2019). Validity and reliability of the FFQ to use among Iranian (Bijani et al., 2018; Malekahmadi et al., 2016) and non‐Iranian elderly (Smith et al., 1998) have been reported by many studies. We calculated the protein content of each food item based on USDA nutrition facts and then categorized them as animal protein (AP), plant protein (PP), and total protein (TP).

### 2.3 Biochemical assessment

A single venous blood sample collected after 12 hours of fasting was used for biochemical assessment. Commercial enzymatic reagents (Pars Azmoon, Tehran, Iran) were used to evaluate serum concentrations of fasting blood sugar (FBS) [glucose oxidase] and triglycerides (TG) [glycerol phosphate oxidase]. An ultrasensitive latex‐enhanced immunoturbidimetric assay (Randox Laboratory Ltd., Belfast, UK) was applied to determine the plasma concentration of high‐sensitive C‐reactive protein (hs‐CRP). Other inflammatory biomarkers were determined using the enzyme‐linked immunosorbent assay (ELISA) method (Boster Biological Technology for IL‐6 and TNF‐a, China).

### 2.4 Anthropometric assessment

Anthropometric indices like body weight, height, and waist circumference were assessed by a trained assistant. A portable digital scale (SECA 813; Seca) was used to measure participant weight (measurement precision of 100 g) when wearing light clothing. To measure participant height, a tape with 0.5 cm of precision was used while participants were asked to stand against a wall in a normal position without moving. For waist measurements, a tape measure within 0.5 cm of precision was used to measure at the middle point between the top of the hips and bottom of the ribs was selected. BMI was calculated as participant's weight (kg) divided by height (m^2^).

### 2.5 Assessment of other covariates

A valid and reliable questionnaire was used to assess the socioeconomic status (SES; Mozaffari et al., 2019). The questionnaire considered occupation, education, vehicle and house ownership, modern household appliances, the number of rooms and family members, and trips through the previous year. For other covariates, a demographic questionnaire was used, which considered age, marital, and smoking status. Participants were asked about chronic diseases such as diabetes, hyperlipidemia, myocardial infarction, hypertension, stroke, angina, and thyroid disease. They were also asked about their drugs such as heart disease drugs, diabetes drugs, and lipid‐lowering and thyroid drugs.

Participants were asked to be seated for about 10 minutes, and blood pressure (BP) was measured twice within a one‐minute interval. Their bladders were emptied. Moreover, no caffeinated beverages consumed and no exercises were done within 1 h before the measurement. The average time for activities was presented as (MET‐h/week).

### 2.6 Statistical analysis

Kolmogorov–Smirnov test and the histogram curves were assessed to determine the normal distributions of the covariates. Participants were categorized based on their dietary intake of animal protein (AP; <34.76, 34.76–47.06, >47.06), plant protein (PP; <21.57, 21.57–27.60, >27.6), and total protein (TP; <66.76, 66.76–90.84, >90.84). General characteristics were described for animal, plant, and total protein turtles. Chi‐squared test was used to test the distribution of elderly men with continuous variables, and one‐way analysis of variance (ANOVA) was used to test the distribution of elderly men with categorical variables within the tertiles of animal, plant and total protein intakes. In addition, one‐way analysis of covariance (ANCOVA) with energy intake adjustment was used to compare the distribution of nutrients and food group intakes across tertiles of animal, plant, and total protein intake. One‐way analysis of covariance (ANCOVA) was also used to compare cardiovascular outcomes and inflammatory parameters across tertiles of animal, protein, and total protein intake in both crude and adjusted models.

To conduct binary logistic regression, participants were categorized for cardiovascular risk factors following the reported cutoffs from the Adult Treatment Panel III (ATP‐III; Maas & Appelman, 2010): WC > 88 cm, BP ≥ 130/85 mmHg, FBS ≥ 110 mg/dL, and TG ≥ 150 mg/dL. For serum hs‐CRP level, previous cutoffs for Iranian elderly were used (hs‐CRP >2 mg/L) (Rashidi Pour Fard et al., 2015). To assess the association between animal, plant, and total protein intake and cardio‐metabolic risk factors, binary logistic regression in crude and two adjusted models were applied. The following confounders were adjusted in the final model: age, marital status, physical activity, socioeconomic status, smoking status, diseases and drugs, body mass index (BMI), fat, and carbohydrate intake. Plant protein was included as an additional cofounder in models with animal protein, and animal protein was included as an additional cofounder in the models with plant protein. Adjusted cofounders were selected based on previous relevant studies as well as univariate models, which were conducted for each outcome, and associations with *p* < .2 were considered as possible cofounders. The first tertiles of dietary intake were the reference group. SPSS software version 26.0 was used to analyze the data, and *p* values < .05 were considered statistically significant.

## 3 RESULTS

Participants' general characteristics across tertiles of AP, PP, and TP are shown in Table 1. Participants in the highest tertile of AP had higher age (*p* = .02), WC (*p* = .001), and BMI (*p* = .03) were more likely to be nonsmokers (*p* = .04) and not consume certain drugs such as lipid‐lowering drugs (*p* = .016) and heart disease drugs (*p* < .001). Participants in the highest tertile of PP had lower age (*p* < .001), lower WC (*p* = .02), and lower weight (*p* = .01). They also were less likely to use certain drugs like antidiabetic drugs (*p* = .007) and heart disease drugs (*p* = .020). In addition, participants who were in the highest tertile of TP were more likely to be nonsmokers (*p* < .001) and not consume certain medications such as antidiabetic drugs (*p* = .015), lipid‐lowering drugs, and heart disease drugs (*p* < .001).

**TABLE 1:** General characteristics of participants across the tertiles of AP, PP, and TP.

Characteristics	AP				PP				TP			
	T1	T2	T3	*p*_value_ ^a^	T1	T2	T3	*p*_value_^a^	T1	T2	T3	*p*_value_^a^
	<34.76	34.76, 47.06	>47.06		<21.57	21.57, 27.60	>27.6		<66.76	66.76, 90.84	>90.84	
*n*	121	117	119		119	122	115		118	120	119	
Age (year)	63.64 ± 5.90	65.63 ± 6.93	65.66 ± 6.53	.022	65.34 ± 7.45	66.65 ± 6.87	62.81 ± 4.09	<.001	65.05 ± 5.98	65.66 ± 7.69	64.18 ± 5.62	.214
Weight (kg)	70.18 ± 10.11	74.50 ± 8.99	71.93 ± 11.13	.005	74.10 ± 9.63	70.27 ± 12.26	72.22 ± 8.00	.015	70.28 ± 10.65	73.34 ± 10.68	72.89 ± 9.15	.045
Height (cm)	169.10 ± 7.04	168.11 ± 6.94	168.71 ± 6.18	.518	170.29 ± 6.95	166.19 ± 7.32	169.54 ± 4.90	<.001	168.83 ± 7.29	167.82 ± 7.50	169.28 ± 5.08	.226
Physical activity (Met/min/week)	0.85 ± 0.10	0.85 ± 0.12	0.84 ± 0.10	.657	0.83 ± 0.10	0.86 ± 0.12	0.85 ± 0.09	.252	0.85 ± 0.10	0.85 ± 0.12	0.84 ± 0.10	.696
BMI (kg/m^2^)	24.77 ± 2.94	25.46 ± 3.24	25.85 ± 3.31	.030	24.94 ± 2.86	24.96 ± 3.23	26.20 ± 3.33	.002	25.14 ± 2.93	24.96 ± 3.15	25.97 ± 3.41	.034
WC (cm)	94.30 ± 11.00	98.27 ± 6.79	95.91 ± 6.76	.001	97.91 ± 6.74	95.37 ± 11.63	95.14 ± 5.83	.022	94.95 ± 11.10	97.25 ± 7.79	96.20 ± 6.00	.117
Marital status												
Married	108 (97.5)	109 (93.2)	117 (98.3)	.075	117 (98.3)	113 (92.6)	114 (98.3)	.025	116 (98.3)	110 (91.7)	118 (99.2)	.003
Single‐divorced	3 (2.5)	8 (6.8)	2 (1.7)		2 (1.7)	9 (7.4)	2 (1.7)		2 (1.7)	10 (8.3)	1 (0.8)	
SES, *n* (%)												
Low	45 (37.2)	46 (39.3)	50 (42.0)	.007	37 (31.1)	60 (49.2)	44 (37.9)	<.001	38 (32.2)	56 (46.7)	47 (39.5)	<.001
Moderate	64 (52.9)	51 (43.6)	39 (32.8)		71 (59.7)	38 (31.1)	45 (38.8)		70 (59.3)	35 (29.2)	49 (41.2)	
High	12 (9.9)	20 (17.1)	30 (25.2)		11 (9.2)	24 (19.7)	27 (23.3)		10 (8.5)	29 (24.2)	23 (19.3)	
Smoking, *n* (%)												
No	93 (76.9)	97 (82.9)	106 (89.1)	.042	91 (76.5)	110 (90.2)	95 (81.9)	.017	83 (70.3)	108 (90)	105 (88.2)	<.001
Yes	28 (23.1)	20 (17.1)	13 (10.9)		28 (23.5)	12 (9.8)	21 (18.1)		35 (29.7)	12 (10)	14 (11.8)	
Disease, *n* (%)												
No	81 (66.9)	79 (67.5)	59 (49.6)	.005	63 (52.9)	89 (73)	67 (57.8)	.004	71 (60.2)	89 (74.2)	59 (49.6)	<.001
Yes	40 (33.1)	38 (32.5)	60 (50.4)		56 (47.1)	33 (27)	49 (42.2)		47 (39.8)	31 (25.8)	60 (50.4)	
Antidiabetic drugs, *n* (%)												
No	89 (73.6)	87 (74.4)	96 (80.7)	.368	97 (81.5)	81 (66.4)	94 (81)	.007	88 (74.6)	83 (69.2)	101 (84.9)	.015
Yes	32 (26.4)	30 (25.6)	23 (19.3)		22 (18.5)	41 (33.6)	22 (19)		30 (25.4)	37 (30.8)	18 (15.1)	
Lipid‐lowering drugs, *n* (%)												
No	96 (79.3)	91 (77.8)	108 (90.8)	.016	103 (86.6)	96 (78.7)	96 (82.8)	.273	104 (88.1)	82 (68.3)	109 (91.6)	<.001
Yes	25 (20.7)	26 (22.2)	11 (9.2)		16 (13.4)	26 (21.3)	20 (17.2)		14 (11.9)	38 (31.7)	10 (8.4)	
Thyroid drugs, *n* (%)												
No	116 (95.9)	114 (97.4)	117 (98.3)	.507	115 (96.6)	119 (97.5)	113 (97.4)	.910	113 (95.8)	120 (100)	114 (95.8)	.074
Yes	5 (4.1)	3 (2.6)	2 (1.7)		4 (3.4)	3 (2.5)	3 (2.6)		5 (4.2)	0 (0)	5 (4.2)	
Heart disease drugs, *n* (%)												
No	103 (85.1)	77 (65.8)	99 (83.2)	<.001	98 (82.4)	85 (69.7)	96 (82.8)	.020	103 (87.3)	79 (65.8)	97 (81.5)	<.001
Yes	18 (14.9)	40 (34.2)	20 (16.8)		21 (17.6)	37 (30.3)	20 (17.2)		15 (12.7)	41 (34.2)	22 (18.5)	

*Note*: *p* value < .05 was considered significant. Values are based on average ± standard deviation or reported percentage. Analysis of variance ANOVA for quantitative data and Chi‐2 test for qualitative data have been used.

Abbreviations: AP, animal protein; BMI,body mass index; PP,plant protein; TP, total protein; WC, waist circumference.

^a^ Calculated by analysis of variance ANOVA for quantitative data and Chi‐2 test for qualitative data.

Dietary intakes of the participants across tertiles of AP, PP, and TP are displayed in Table 2. Participants in the highest tertile of AP had higher intake of energy (*p* = .001), phosphorus (*p* < .001), potassium (*p* < .001), calcium (*p* < .001), magnesium (*p* < .001), sodium (*p* < .001), zinc (*p* = .001), iron (*p* = .01), fruits (*p* = .03), and dairy products (*p* = .001) but lower intake of carbohydrates (*p* < .001), fiber (*p* < .001) and n‐3 fatty acids (*p* < .001). Participants in the highest tertile of PP had higher intake of energy (*p* < .001), carbohydrates (*p* = .01), fiber (*p* = .001), magnesium (*p* < .001), iron (*p* < .001), n‐3 fatty acids (*p* = .001), fruits (P < .001) and vegetables (*p* < .001) and lower intake of phosphorus (*p* = .001), calcium (*p* = .001), and dairy products (*p* < .001). Participants included in the highest tertile of TP had higher intake of energy (*p* = .001), fiber (*p* = .001), phosphorus (*p* < .001), potassium (*p* < .001), calcium (*p* < .001), magnesium (*p* < .001), sodium (*p* = .01), zinc (*p* < .001), iron (*p* < .001), n‐3 fatty acids (*p* = .001), fruits (*p* < .001), vegetables (*p* < .001) and dairy products (*p* < .001), and lower intake of fat (*p* < .001).

**TABLE 2:** Energy‐adjusted dietary intakes across tertiles of AP, PP, and TP.

Food groups	AP (g/day)				PP (g/day)				TP (g/day)			
	T1	T2	T3	*p*_value_ ^a^	T1	T2	T3	*p*_value_ ^a^	T1	T2	T3	*p*_value_ ^a^
	<34.76	34.76, 47.06	>47.06		<21.57	21.57, 27.60	>27.6		<66.76	66.76, 90.84	>90.84	
*N*	121	117	119		119	122	115		118	120	118	
Nutrients^b^												
Energy	1782.76 ± 36.88	2065.04.0 ± 44.63	2621.66 ± 53.48	<.001	1685.85 ± 41.29	2134.01 ± 35.54	2658.39 ± 49.18	<.001	1664.15 ± 28.96	2176.29 ± 45.38	2619.97 ± 50.14	<.001
Carbohydrate	339.63 ± 4.09	342.21 ± 3.83	323.47 ± 4.32	.006	324.01 ± 4.40	339.79 ± 3.74	341.66 ± 4.55	.014	339.50 ± 4.49	336.61 ± 3.80	329.23 ± 4.44	.309
Fat	63.29 ± 1.62	60.55 ± 1.51	58.89 ± 1.71	.211	61.99 ± 1.74	60.66 ± 1.48	60.14 ± 1.80	.783	63.58 ± 1.70	65.17 ± 1.44	53.97 ± 1.68	<.001
Fiber	11.90 ± 0.37	13.26 ± 0.34	10.85 ± 0.39	<.001	9.10 ± 0.35	11.90 ± 0.30	15.10 ± 0.37	<.001	10.33 ± 0.38	13.99 ± 0.32	11.64 ± 0.37	<.001
Phosphorus	1357.9 ± 39.59	1622.2 ± 37.06	2422.74 ± 41.85	<.001	1986.85 ± 56.90	1705.77 ± 48.37	1699.54 ± 58.81	.001	1344.76 ± 40.15	1563.06 ± 33.99	2489.31 ± 39.64	<.001
Potassium	3371 ± 84.54	4053.77 ± 79.14	4785.38 ± 89.36	<.001	4017.05 ± 103.53	3961 ± 88.02	4222.92 ± 107.02	.172	3213.06 ± 85.29	3957.52 ± 72.20	5023.85 ± 84.22	<.001
Calcium	1043.41 ± 51.88	1284.55 ± 48.56	2189.91 ± 54.84	<.001	1732.71 ± 69.05	1393.35 ± 58.70	1380.64 ± 71.38	.001	1021.76 ± 49.90	1173.57 ± 42.24	2318.29 ± 49.28	<.001
Magnesium	273.87 ± 6.84	318.28 ± 6.40	367.38 ± 7.23	<.001	301.33 ± 7.89	309.68 ± 6.71	348.57 ± 8.16	<.001	255.11 ± 6.76	313.08 ± 5.72	390.28 ± 6.68	<.001
Sodium	6.19 ± 1.92	7.55 ± 1.80	14.80 ± 2.03	.011	8.01 ± 2.07	12.92 ± 1.76	7.38 ± 2.14	.056	5.79 ± 2.08	7.78 ± 1.76	14.93 ± 2.06	.010
Zinc	7.2 ± 0.29	8.4 ± 0.27	11.9 ± 0.31	.001	9.9 ± 0.36	8.9 ± 0.30	8.9 ± 0.37	.08	6.7 ± 0.28	8 ± 0.24	12.9 ± 0.28	<.001
Iron	11.8 ± 0.33	13.1 ± 3.0	12.3 ± 0.31	.01	10 ± 0.29	11.9 ± 0.24	15.4 ± 0.30	<.001	10.9 ± 0.33	13.5 ± 0.28	12.8 ± 0.32	<.001
Folate	370.02 ± 10.11	402.88 ± 9.47	406.84 ± 10.69	.027	346.32 ± 10.43	386.66 ± 8.87	448.09 ± 10.78	<.001	338.92 ± 10.59	407.70 ± 8.97	432.19 ± 10.46	<.001
n‐3 fatty acids	0.37 ± 0.02	0.39 ± 0.02	0.28 ± 0.02	<.001	0.22 ± 0.02	0.35 ± 0.02	0.46 ± 0.02	.001	0.27 ± 0.02	0.47 ± 0.02	0.29 ± 0.02	.001
Food groups^b^												
Grains	328.07 ± 13.96	343.22 ± 13.07	329.34 ± 14.76	.657	315.13 ± 14.95	338.66 ± 12.71	346.94 ± 15.46	.368	351.81 ± 15.12	317.75 ± 12.80	331.11 ± 14.93	.228
Fruits	425.18 ± 17.83	486.48 ± 16.69	456.49 ± 18.85	.037	327.94 ± 17.64	490.37 ± 15.00	551.14 ± 18.23	<.001	385.28 ± 19.04	493.44 ± 16.12	487.77 ± 18.80	<.001
Vegetables	384.13 ± 16.64	409.00 ± 15.58	395.92 ± 17.59	.531	338.31 ± 17.49	400.99 ± 14.87	451.07 ± 18.08	<.001	330.41 ± 17.37	455.86 ± 14.70	401.36 ± 17.15	<.001

*Note*: *p* Value < .05 was considered significant. All the variables, except energy, adjusted for energy intake.

Abbreviations: AP, animal protein; PP, plant protein; TP, total protein.

^a^ Calculated by analysis of variance (ANOVA) for energy intake and multivariate analysis of covariance (ANCOVA) for other dietary variables.

^b^ Mean ± SE.

Cardiovascular risk factors and inflammatory factors across tertiles of AP, PP, and TP are presented in Table 3. Participants in the highest tertile of AP had higher WC (*p* < .001) and lower SBP (*p* = .003), FBS (*p* = .001) and LDL/HDL (*p* = .012). Participants in the highest tertile of PP had lower WC (*p* = .033), FBS (*p* = .049), LDL/HDL (*p* = .047), and Hs‐CRP (*p* = .046). Participants in the highest tertile of TP had higher WC (*p* < .001) and lower SBP (*p* = .006), FBS (*p* = .016), LDL/HDL (*p* = .003), and Hs‐CRP (*p* = .001).

**TABLE 3:** Cardiovascular risk factors across tertiles of AP, PP, and TP.

Characteristics	AP				PP				TP			
	T1	T2	T3	*p*_value_	T1	T2	T3	*p*_value_	T1	T2	T3	*p*_value_
	<34.76	34.76, 47.06	>47.06		<21.57	21.57, 27.60	>27.6		<66.76	66.76, 90.84	>90.84	
*n*	121	117	119		119	122	115		118	120	118	
WC												
Crude	94.31 ± 0.77	98.27 ± 0.78	95.91 ± 0.77	.001	97.91 ± 0.78	95.37 ± 0.77	95.14 ± 0.79	.022	94.95 ± 0.79	97.25 ± 0.78	96.20 ± 0.78	.117
Model 1	93.78 ± 0.82	98.02 ± 0.76	96.71 ± 0.87	.001	98.10 ± 0.89	95.84 ± 0.77	94.45 ± 0.96	.040	93.23 ± 0.90	97.79 ± 0.76	97.38 ± 0.88	.001
Model 2	93.46 ± 0.80	99.46 ± 0.75	95.60 ± 0.87	<.001	97.98 ± 0.96	96.71 ± 0.75	93.61 ± 1.05	.033	91.85 ± 0.91	99.57 ± 0.77	96.95 ± 0.92	<.001
SBP												
Crude	13.13 ± 0.15	12.91 ± 0.15	12.63 ± 0.15	.057	12.62 ± 0.15	13.14 ± 0.15	12.91 ± 0.15	.043	13.02 ± 0.15	12.93 ± 0.15	12.72 ± 0.15	.340
Model 1	13.36 ± 0.15	12.87 ± 0.14	12.44 ± 0.16	.001	12.62 ± 0.17	13.03 ± 0.14	13.03 ± 0.18	.147	13.32 ± 0.17	12.79 ± 0.14	12.58 ± 0.17	.015
Model 2	13.35 ± 0.16	12.83 ± 0.15	12.48 ± 0.17	.003	12.86 ± 0.18	13.08 ± 0.14	12.73 ± 0.20	.273	13.43 ± 0.18	12.68 ± 0.16	12.58 ± 0.18	.006
DBP												
Crude	7.76 ± 0.07	7.81 ± 0.07	7.87 ± 0.07	.569	7.74 ± 0.07	7.78 ± 0.07	7.93 ± 0.07	.176	7.70 ± 0.07	7.70 ± 0.07	7.99 ± 0.07	.012
Model 1	7.87 ± 0.07	7.82 ± 0.07	7.74 ± 0.08	.549	7.88 ± 0.08	7.83 ± 0.07	7.73 ± 0.09	.503	7.88 ± 0.08	7.72 ± 0.07	7.85 ± 0.08	.234
Model 2	7.91 ± 0.08	7.80 ± 0.07	7.73 ± 0.08	.335	7.94 ± 0.09	7.84 ± 0.07	7.65 ± 0.10	.187	7.89 ± 0.09	7.69 ± 0.08	7.86 ± 0.09	.151
FBS												
Crude	107.84 ± 1.87	100.80 ± 1.91	100.50 ± 1.89	.008	102.92 ± 1.92	103.24 ± 1.89	103.11 ± 1.94	.993	104.86 ± 1.90	105.48 ± 1.89	98.92 ± 1.90	.027
Model 1	108.27 ± 1.99	100.91 ± 1.85	100.04 ± 2.10	.010	100.88 ± 2.17	102.18 ± 1.87	106.45 ± 2.33	.275	106.37 ± 2.18	104.52 ± 1.85	98.47 ± 2.14	.045
Model 2	108.79 ± 1.90	98.97 ± 1.77	101.69 ± 2.06	.001	105.38 ± 2.30	99.59 ± 1.77	104.83 ± 2.48	.049	109.01 ± 2.22	100.94 ± 1.91	99.70 ± 2.20	.016
TG												
Crude	130.46 ± 3.43	128.20 ± 3.48	132.93 ± 3.45	.628	136.31 ± 3.43	124.00 ± 3.38	131.51 ± 3.47	.037	133.17 ± 3.45	124.76 ± 3.42	133.77 ± 3.44	.117
Model 1	130.68 ± 3.77	128.50 ± 3.50	132.06 ± 3.97	.787	138.41 ± 4.01	125.11 ± 3.47	127.79 ± 4.32	.039	134.14 ± 4.09	125.17 ± 3.46	132.04 ± 4.01	.175
Model 2	131.67 ± 3.89	130.16 ± 3.57	129.80 ± 4.18	.946	132.72 ± 4.14	126.20 ± 3.53	129.83 ± 4.39	.219	135.38 ± 4.29	125.06 ± 3.67	131.35 ± 4.44	.171
HDL												
Crude	49.02 ± 0.80	49.49 ± 0.81	49.76 ± 0.80	.800	49.36 ± 0.80	50.70 ± 0.79	48.14 ± 0.81	.077	48.36 ± 0.80	50.59 ± 0.80	49.29 ± 0.80	.144
Model 1	49.60 ± 0.87	49.44 ± 0.81	49.18 ± 0.92	.955	50.10 ± 0.93	50.60 ± 0.80	47.42 ± 1.00	.059	48.50 ± 0.95	50.48 ± 0.80	49.23 ± 0.93	.242
Model 2	50.12 ± 0.90	48.96 ± 0.82	49.18 ± 0.96	.617	50.13 ± 0.93	50.43 ± 0.80	47.62 ± 1.00	.103	49.04 ± 0.98	50.41 ± 0.83	48.80 ± 1.02	.370
LDL												
Crude	98.35 ± 1.91	93.85 ± 1.95	92.91 ± 1.93	.103	100.24 ± 1.91	91.86 ± 1.89	93.11 ± 1.94	.004	98.50 ± 1.94	93.39 ± 1.92	93.34 ± 1.93	.097
Model 1	97.47 ± 2.09	93.50 ± 1.94	93.97 ± 2.20	.347	99.72 ± 2.23	92.75 ± 1.93	92.51 ± 1.93	.044	96.74 ± 2.28	94.15 ± 1.93	94.14 ± 2.24	.676
Model 2	98.47 ± 2.04	95.23 ± 1.87	91.35 ± 2.18	.107	95.81 ± 2.18	94.99 ± 1.84	94.35 ± 2.28	.921	96.55 ± 2.26	97.39 ± 1.90	91.17 ± 2.33	.138
TC												
Crude	178.93 ± 2.32	176.12 ± 2.36	174.74 ± 2.34	.433	183.86 ± 2.29	169.58 ± 2.26	176.58 ± 2.32	<.001	179.82 ± 2.34	172.33 ± 2.32	177.75 ± 2.33	.065
Model 1	177.59 ± 2.51	175.89 ± 2.32	176.14 ± 2.64	.875	183.28 ± 2.64	171.21 ± 2.29	175.26 ± 2.85	.002	176.79 ± 2.72	173.68 ± 2.30	179.23 ± 2.67	.260
Model 2	180.42 ± 2.33	177.78 ± 2.14	171.38 ± 2.50	.058	178.89 ± 2.50	174.89 ± 2.13	175.96 ± 2.65	.482	178.63 ± 2.60	177.38 ± 2.22	173.67 ± 2.69	.483
LDL/HDL												
Crude	2.08 ± 0.04	1.93 ± 0.04	1.87 ± 0.04	.003	2.10 ± 0.04	1.81 ± 0.04	1.97 ± 0.04	<.001	2.11 ± 0.04	1.88 ± 0.04	1.90 ± 0.04	<.001
Model 1	2.03 ± 0.05	1.93 ± 0.04	1.92 ± 0.05	.203	2.07 ± 0.05	1.84 ± 0.04	1.97 ± 0.05	.002	2.08 ± 0.05	1.90 ± 0.04	1.90 ± 0.05	.024
Model 2	2.07 ± 0.05	1.97 ± 0.04	1.84 ± 0.05	.012	2.03 ± 0.05	1.87 ± 0.04	1.99 ± 0.05	.047	2.12 ± 0.05	1.95 ± 0.04	1.81 ± 0.05	.003
Hs‐CRP												
Crude	3.48 ± 0.56	2.68 ± 0.57	2.11 ± 0.56	.223	3.45 ± 0.56	1.95 ± 0.55	2.90 ± 0.57	.160	3.48 ± 0.56	2.83 ± 0.56	1.96 ± 0.56	.160
Model 1	3.55 ± 0.60	2.84 ± 0.56	1.86 ± 0.63	.208	4.59 ± 0.65	2.27 ± 0.55	1.39 ± 0.69	.007	4.54 ± 0.64	2.85 ± 0.55	0.88 ± 0.63	.002
Model 2	3.83 ± 0.60	2.68 ± 0.56	1.74 ± 0.64	.099	4.10 ± 0.71	1.89 ± 0.55	2.29 ± 0.77	.046	4.74 ± 0.58	2.90 ± 0.68	0.64 ± 0.68	.001
Fibrinogen (mg/dL)												
Crude	281.99 ± 4.24	277.32 ± 4.31	264.46 ± 4.27	.011	280.31 ± 4.31	269.98 ± 4.26	273.65 ± 4.37	.227	283.36 ± 4.31	272.85 ± 4.27	267.73 ± 4.29	.033
Model 1	274.93 ± 4.57	277.18 ± 4.25	272.37 ± 4.82	.761	269.89 ± 4.91	272.97 ± 4.23	281.89 ± 5.28	.318	273.31 ± 4.99	276.04 ± 4.23	275.09 ± 4.89	.919
Model 2	276.02 ± 4.67	277.46 ± 4.27	270.97 ± 5.06	.644	270.12 ± 5.01	271.45 ± 4.30	283.25 ± 5.28	.201	274.30 ± 5.28	275.69 ± 4.48	274.45 ± 5.45	.974
IL‐6												
Crude	6.59 ± 0.07	6.41 ± 0.07	6.43 ± 0.07	.132	6.47 ± 0.07	6.57 ± 0.07	6.39 ± 0.07	.196	6.61 ± 0.07	6.45 ± 0.07	6.37 ± 0.07	.037
Model 1	6.57 ± 0.07	6.40 ± 0.07	6.45 ± 0.08	.207	6.47 ± 0.08	6.56 ± 0.07	6.39 ± 0.09	.305	6.64 ± 0.08	6.42 ± 0.07	6.36 ± 0.08	.054
Model 2	6.57 ± 0.08	6.41 ± 0.07	6.44 ± 0.08	.277	6.42 ± 0.09	6.55 ± 0.07	6.45 ± 0.09	.402	6.62 ± 0.09	6.45 ± 0.08	6.35 ± 0.09	.142
TNF‐α (pg/mL)												
Crude	0.73 ± 0.01	0.72 ± 0.01	0.73 ± 0.01	.561	0.73 ± 0.01	0.72 ± 0.01	0.73 ± 0.01	.539	0.73 ± 0.01	0.73 ± 0.01	0.72 ± 0.01	.634
Model 1	0.73 ± 0.01	0.72 ± 0.01	0.73 ± 0.01	.513	0.72 ± 0.01	0.72 ± 0.01	0.74 ± 0.01	.365	0.72 ± 0.01	0.73 ± 0.01	0.72 ± 0.01	.582
Model 2	0.73 ± 0.01	0.72 ± 0.01	0.74 ± 0.01	.212	0.73 ± 0.01	0.72 ± 0.01	0.74 ± 0.01	.252	0.73 ± 0.01	0.73 ± 0.01	0.73 ± 0.01	.886
ALT												
Crude	20.99 ± 1.35	20.21 ± 1.37	18.84 ± 1.36	.526	19.42 ± 1.36	20.48 ± 1.35	20.15 ± 1.38	.852	19.99 ± 1.37	20.72 ± 1.36	19.34 ± 1.36	.771
Model 1	21.74 ± 1.43	20.52 ± 1.33	17.47 ± 1.51	.162	20.91 ± 1.54	20.66 ± 1.34	18.13 ± 1.66	.476	22.39 ± 1.56	20.36 ± 1.32	17.02 ± 1.53	.085
Model 2	21.39 ± 1.44	21.20 ± 1.33	17.16 ± 1.54	.126	19.45 ± 1.71	19.43 ± 1.34	20.94 ± 1.85	.818	21.08 ± 1.63	20.99 ± 1.39	17.69 ± 1.65	.310
AST												
Crude	21.76 ± 1.25	21.36 ± 1.27	21.51 ± 1.26	.975	22.40 ± 1.26	20.07 ± 1.24	22.22 ± 1.27	.339	21.30 ± 1.27	21.77 ± 1.26	21.56 ± 1.26	.476
Model 1	21.83 ± 1.34	21.65 ± 1.24	21.06 ± 1.41	.931	24.36 ± 1.42	20.53 ± 1.23	19.61 ± 1.53	.072	23.13 ± 1.45	21.68 ± 1.23	19.74 ± 1.42	.331
Model 2	22.33 ± 1.35	22.18 ± 1.25	20.02 ± 1.45	.502	23.65 ± 1.60	19.56 ± 1.24	21.35 ± 1.72	.105	22.93 ± 1.52	22.53 ± 1.30	19.08 ± 1.54	.210

*Note*: *p* Value < .05 was considered significant. Values are based on Mean ± SE. Crude: Not adjusted for any variables. Model 1: The model was adjusted for age, energy intake, marital status, socioeconomic status, physical activity, smoking, and BMI. Model 2: Model 1 + diseases and drugs, fiber, total fat, and total carbohydrate.

Abbreviations: AP, animal protein; DBP, diastolic blood pressure; FBS, fasting blood sugar; HDL‐C, high‐density lipoprotein cholesterol; hs‐CRP, high‐sensitive C‐reactive protein; IL‐6, interleukin‐6; LDL‐C, low‐density lipoprotein cholesterol; PP, plant protein; SBP, systolic blood pressure; TC, total cholesterol; TG, triglyceride; TNF‐α, tumor necrosis factor‐alpha; TP, total protein; WC, waist circumference.

Odds ratios (OR) and 95% confidence intervals (CI) for cardiovascular risk factors and Hs‐CRP are shown in Table 4. In the final model which is adjusted for all the considered cofounders, a significant inverse association was found between AP and SBP (OR: 0.62; 95% CI: 0.42, 0.91; *p*trend = .014). There were significant inverse association between PP and WC (OR: 0.34; 95% CI: 0.17, 0.68; *p*trend < .001), FBS (OR: 0.51; 95% CI: 0.29, 0.89; *p*trend = .018), and Hs‐CRP (OR: 0.39; 95% CI: 0.21, 0.70; *p*trend = .002). Moreover, significant inverse associations were also found between TP and SBP (OR: 0.54; 95% CI: 0.33, 0.86; *p*trend = .010) and TP and Hs‐CRP (OR: 0.50; 95% CI: 0.28, 0.88; *p*trend = .015).

**TABLE 4:** Multivariate adjusted odds ratios and 95% confidence intervals for blood glucose, lipid profile, and inflammatory factors across tertiles of AP, PP, and TP.

	AP (g/day)				PP (g/day)				TP (g/day)			
Range	<34.76	34.76, 47.06	>47.06	*p* ^a^	<21.57	21.57, 27.60	>27.6	*p* ^a^	<66.76	66.76, 90.84	>90.84	*p* ^a^
*n*	121	117	119		119	122	115		118	120	118	
	T1	T2	T3		T1	T2	T3		T1	T2	T3	
WC												
Crude	1.00	4.95 (2.07–11.83)	0.97 (0.53–1.75)	.927	1.00	0.18 (0.07–0.42)	0.21 (0.09–0.52)	.001	1.00	0.87 (0.45–1.66)	1.06 (0.54–2.08)	.866
Model 1	1.00	4.79 (1.94–11.81)	0.94 (0.43–2.05)	.906	1.00	0.13 (0.05–0.34)	0.12 (0.04–0.36)	<.001	1.00	1.11 (0.52–2.35)	1.44 (0.59–3.49)	.417
Model 2	1.00	9.46 (3.02–29.66)	0.40 (0.14–1.13)	.099	1.00	0.17 (0.06–0.52)	0.10 (0.02–0.41)	**<.001**	1.00	1.98 (0.77–5.12)	0.77 (0.24–2.49)	.691
FBS												
Crude	1.00	0.98 (0.57–1.67)	1.12 (0.66–1.90)	.680	1.00	1.07 (0.63–1.82)	0.95 (0.55–1.64)	.861	1.00	1.21 (0.71–2.06)	0.90 (0.52–1.56)	.716
Model 1^b^	1.00	0.97 (0.54‐1.75)	1.03 (0.51–2.05)	.945	1.00	0.93 (0.51–1.70)	1.06 (0.49–2.27)	.917	1.00	0.91 (0.48–1.70)	0.70 (0.33–1.48)	.341
Model 2^c^	1.00	0.53 (0.25‐1.14)	1.31 (0.54–3.20)	.762	1.00	0.29 (0.13–0.67)	0.27 (0.09–0.85)	**.018**	1.00	0.20 (0.08–0.52)	0.44 (0.16–1.23)	.113
Hypertriglyceridemia												
Crude	1.00	1.14 (0.65–2.00)	0.55 (0.29–1.03)	.073	1.00	1.53 (0.84–2.78)	1.41 (0.77–2.61)	.273	1.00	2.48 (1.38–4.45)	0.85 (0.44–1.65)	.678
Model 1	1.00	1.11 (0.61–2.02)	0.42 (0.19–0.92)	.051	1.00	1.88 (0.97–3.65)	1.50 (0.64–3.52)	.295	1.00	2.91 (1.48–5.73)	0.83 (0.35–1.97)	.630
Model 2	1.00	1.10 (0.54–2.23)	0.39 (0.16–0.95)	.056	1.00	1.38 (0.64–2.98)	0.97 (0.33–2.88)	.961	1.00	2.86 (1.25–6.55)	0.92 (0.33–2.57)	.814
Hypercholesterolemia												
Crude	1.00	0.99 (0.54–1.83)	0.36 (0.17–0.76)	.010	1.00	1.70 (0.87–3.33)	1.26 (0.62–2.55)	.527	1.00	2.95 (1.54–5.67)	0.59 (0.26–1.36)	.310
Model 1	1.00	1.02 (0.53–1.96)	0.33 (0.13–0.84)	.038	1.00	2.76 (1.28–5.96)	2.55 (0.93–6.97)	.055	1.00	4.28 (1.99–9.20)	0.84 (0.29–2.39)	.899
Model 2	1.00	1.15 (0.48–2.76)	0.36 (0.11–1.21)	.161	1.00	1.56 (0.61–4.00)	0.98 (0.25–3.88)	.993	1.00	4.86 (1.82–12.97)	1.09 (0.23–5.20)	.367
SBP												
Crude	1.00	0.87 (0.53–1.45)	0.52 (0.31–0.87)	.013	1.00	1.29 (0.78–2.15)	1.33 (0.79–2.23)	.276	1.00	0.76 (0.46–1.27)	0.56 (0.34–0.95)	.031
Model 1	1.00	0.66 (0.37–1.17)	0.35 (0.18–0.70)	.003	1.00	1.26 (0.69–2.28)	1.37 (0.64–2.93)	.409	1.00	0.44 (0.24–0.83)	0.27 (0.13–0.59)	.001
Model 2	1.00	0.61 (0.33–1.12)	0.39 (0.18–0.83)	**.014**	1.00	1.02 (0.52–1.97)	0.67 (0.26–1.74)	.459	1.00	0.37 (0.16–0.72)	0.29 (0.11–0.74)	**.010**
DBP												
Crude	1.00	0.67 (0.39–1.16)	0.99 (0.57–1.74)	.970	1.00	1.00 (0.59–1.70)	1.47 (0.84–2.58)	.186	1.00	0.92 (0.54–1.56)	1.49 (0.85–2.61)	.176
Model 1	1.00	0.55 (0.29–1.02)	0.68 (0.32–1.44)	.246	1.00	1.13 (0.60–2.14)	1.38 (0.61–3.15)	.445	1.00	0.97 (0.49–1.90)	1.19 (0.54–2.66)	.655
Model 2	1.00	0.32 (0.15–0.66)	0.53 (0.22–1.24)	.077	1.00	1.18 (0.56–2.51)	0.70 (0.24–2.04)	.634	1.00	0.58 (0.25–1.35)	0.73 (0.28–1.90)	.540
HS‐CRP												
Crude	1.00	0.99 (0.59–1.65)	1.52 (0.91–2.53)	.109	1.00	0.84 (0.50–1.39)	1.23 (0.73–2.05)	.443	1.00	0.88 (0.52–1.46)	1.23 (0.73–2.04)	.434
Model 1	1.00	0.73 (0.40–1.31)	0.77 (0.39–1.53)	.414	1.00	0.46 (0.24–0.87)	0.16 (0.07–0.38)	<.001	1.00	0.50 (0.26–0.95)	0.31 (0.14–0.68)	.003
Model 2	1.00	0.63 (0.30–1.31)	0.56 (0.23–1.38)	.186	1.00	0.41 (0.19–0.88)	0.15 (0.04–0.48)	**.002**	1.00	0.50 (0.21–1.20)	0.25 (0.08–0.77)	**.015**

*Note*: Data are OR (95% CI). Bold indicates statistical significant value (*p* < .05).

^a^ *p* For trend Calculate by logistic regression.

^b^ Adjusted for age, smoking, physical activity, socioeconomic status, marital status, and energy.

^c^ Adjusted for age, smoking, physical activity, socioeconomic status, marital status, energy, disease, antidiabetic drugs, thyroid drugs, and heart disease drugs.

As a significant inverse association between AP and SBP was found, we conducted another logistic regression to find the potential reason. Odds ratios (OR) and 95% confidence intervals (CI) for SBP and DBP across tertiles of different animal protein groups are presented in Table 5 and Table 6. We found that the consumption of dairy products had a significant inverse association with SBP (OR: 0.61; 95% CI: 0.41, 0.91; *p*trend = .017). In addition, there was a significant inverse association between egg and DBP (OR: 0.57; 95% CI: 0.37, 0.87; *p*trend = .010).

**TABLE 5:** Multivariate adjusted odds ratios and 95% confidence intervals for blood pressure across tertiles of eggs, poultry, and fish.

	Eggs (g/day)				Poultry (g/day)				Fish (g/day)			
Range	<0.99	0.99	>0.99	*p* ^a^	<2.92	2.92, 7.08	>7.08	*p* ^a^	<5.84	5.84, 7.29	>7.29	*p* ^a^
*n*	105	179	73		154	51	152		164	95	98	
	T1	T2	T3		T1	T2	T3		T1	T2	T3	
SBP												
Crude	1.00	2.40 (1.46–3.95)	0.85 (0.45–1.61)	.927	1.00	1.41 (0.75–2.66)	1.20 (0.76–1.89)	.426	1.00	0.64 (0.38–1.08)	0.92 (0.56–1.52)	.581
Model 1	1.00	2.69 (1.55–4.67)	0.90 (0.44–1.83)	.736	1.00	1.43 (0.73–2.82)	1.12 (0.68–1.85)	.653	1.00	0.86 (0.48–1.53)	1.04 (0.60–1.80)	.940
Model 2	1.00	2.88 (1.56–5.32)	0.72 (0.33–1.56)	.817	1.00	1.63 (0.78–3.41)	1.20 (0.67–2.13)	.507	1.00	1.00 (0.53–1.86)	1.35 (0.73–2.48)	.373
DBP												
Crude	1.00	1.20 (0.71–2.03)	0.65 (0.35–1.21)	.242	1.00	3.56 (1.57–8.10)	1.61 (1.00–2.60)	.045	1.00	0.75 (0.43–1.30)	0.58 (0.34–0.99)	.046
Model 1^b^	1.00	0.99 (0.54‐1.80)	0.43 (0.21–0.88)	.039	1.00	3.30 (1.35–8.03)	1.63 (0.94–2.82)	.068	1.00	0.92 (0.48–1.75)	0.59 (0.32–1.08)	.100
Model 2^c^	1.00	0.70 (0.34‐1.41)	0.31 (0.13–0.73)	**.010**	1.00	4.67 (1.73–12.64)	1.13 (0.59–2.17)	.573	1.00	1.23 (0.57–2.63)	1.01 (0.49–2.08)	.941

*Note*: Data are OR (95% CI). Bold indicates statistical significant value (*p* < .05).

^a^ *p* For trend Calculate by logistic regression.

^b^ Adjusted for age, smoking, physical activity, socioeconomic status, marital status, and energy.

^c^ Adjusted for age, smoking, physical activity, socioeconomic status, marital status, energy, disease, antidiabetic drugs, thyroid drugs and heart disease drugs.

**TABLE 6:** Multivariate adjusted odds ratios and 95% confidence intervals for blood pressure across tertiles of red meat, processed meat, and dairy products.

	Red meat (g/day)				Processed meat (g/day)				Dairy products			
Range	<1.49	1.49, 3.49	>3.49	*p* ^a^	<0.20	0.20	>0.20	*p* ^a^	<18.62	18.62, 25.29	>25.29	*p* ^a^
*n*	127	112	118		231	8	118		118	118	121	
	T1	T2	T3		T1	T2	T3		T1	T2	T3	
SBP												
Crude	1.00	0.89 (0.53–1.48)	0.84 (0.50–1.38)	.485	1.00	0.17 (0.02–1.44)	1.18 (0.76–1.84)	.521	1.00	1.03 (0.62–1.72)	0.70 (0.42–1.17)	.173
Model 1	1.00	0.88 (0.49–1.57)	1.05 (0.57–1.95)	.879	1.00	0.10 (0.01–0.84)	1.26 (0.77–2.05)	.426	1.00	0.80 (0.44–1.47)	0.44 (0.22–0.85)	.017
Model 2	1.00	0.76 (0.39–1.49)	1.06 (0.53–2.13)	.825	1.00	0.11 (0.01–1.03)	1.30 (0.77–2.22)	.395	1.00	0.63 (0.32–1.26)	0.39 (0.17–0.88)	**.017**
DBP												
Crude	1.00	1.20 (0.68–2.10)	0.73 (0.43–1.25)	.261	1.00	0.47 (0.11–1.92)	1.07 (0.66–1.72)	.839	1.00	0.65 (0.38–1.10)	1.79 (0.99–3.21)	.064
Model 1^b^	1.00	1.11 (0.57‐2.14)	0.49 (0.25–0.97)	.040	1.00	0.11 (0.02–0.52)	0.92 (0.53–1.60)	.675	1.00	0.71 (0.37–1.36)	1.08 (0.52–2.26)	.971
Model 2^c^	1.00	1.13 (0.51‐2.49)	0.52 (0.23–1.20)	.101	1.00	0.20 (0.03–1.30)	1.43 (0.75–2.74)	.382	1.00	0.44 (0.20–0.96)	0.84 (0.34–2.08)	.383

*Note*: Data are OR (95% CI). Bold indicates statistical significant value (*p* < .05).

^a^ *p* For trend Calculate by logistic regression.

^b^ Adjusted for age, smoking, physical activity, socioeconomic status, marital status, and energy.

^c^ Adjusted for age, smoking, physical activity, socioeconomic status, marital status, energy, disease, antidiabetic drugs, thyroid drugs, and heart disease drugs.

## 4 DISCUSSION

The present study showed that the total amount and sources of protein may have effective roles on cardiovascular outcomes and inflammatory factors as higher intake of protein had a significant inverse association with SBP and Hs‐CRP. In addition, higher intake of PP was significantly associated with lower WC, FBS, and Hs‐CRP. Higher intake of AP was associated with lower SBP, and the association was due to higher intake of dairy products (Table 6).

Associations between the amount and source of protein intake and cardiovascular outcomes and inflammatory factors have been explored in studies that were mostly conducted in adults; a tape within 0.5 cm of precision was used but not specific to the elderly. Available evidence about the association between diet and disease in young adults is not appropriate for the elderly because of the differences in distribution and quantity of body fat mass (Guarner & Rubio‐Ruiz, 2015).

In the present study, a significant positive association between higher dairy intake and SBP was found. In line with our finding, Hidayat et al. (2017) conducted a meta‐analysis about the effects of milk proteins on blood pressure and found that milk protein significantly decreased SBP by −3.33 mm Hg. The possible mechanism for the hypotensive effects of milk proteins may be the inhibition of angiotensin‐converting enzyme activity (Fekete et al., 2013; Nonaka et al., 2014; Pal & Radavelli‐Bagatini, 2013), which catalyzes the conversion of angiotensin I to angiotensin II and leads to arterial vasodilation. Lactokinins and casokininsare angiotensin‐converting enzyme inhibitory peptides present in whey and casein (proteins of milk), respectively (FitzGerald et al., 2004; FitzGerald & Meisel, 2000). In addition, the amino acid content of dairy products may be another possible factor responsible for their hypotensive effects (Jennings et al., 2015; Poggiogalle et al., 2019; Teymoori et al., 2017). According to Landi et al. (2021), the most abundant amino acids in milk are glutamic acid and tyrosine. In a study conducted by Stamler et al. (2009), a significant relationship between dietary glutamic acid and decreased blood pressure was found. Glutamate is included in the glutathione molecule, which has antioxidant activity and may play a significant role in blood pressure homeostasis (Vasdev et al., 2006). Moreover, Altorf‐van der Kuil et al. (2013) found that higher intake of was tyrosine related to a 2.4 mm Hg lower SBP. Tyrosine can be a precursor for norepinephrine synthesis and modulate norepinephrine levels, which then has effects on the sympathetic tone in the vasculature (Siomkajło et al., 2017).

In a present study, we found a significant inverse association between higher intake of PP and WC, FBS, and Hs‐CRP. In accordance with our findings, Park et al. (2018) conducted a study on Korean elders and found a significant inverse association between consuming plant protein and WC. In another study conducted by Lin et al. (2011) in Belgium, in both males and females, a significant inverse association between plant protein intake and WC was found. Furthermore, in a cohort study by Shang et al. (2017) also found an inverse association between plant protein intake and WC. In another cohort study by Hruby and Jacques (2018), a favorable association between PP intake and WC was found. However, in another study conducted with both men and women, Halkjær et al. (2011) found no significant association between plant protein intake and WC. In line with our findings of lowered FBS by PP intake, Comerford and Pasin (2016) found that higher intake of plant protein was significantly associated with a decreased risk of type 2 diabetes. Another study indicated that replacing sources of animal with plant protein may improve glycemic control in people with diabetes (Viguiliouk et al., 2015). Plant‐based proteins are high in amino acids like arginine and pyruvigenic amino acids (glycine, alanine, and serine) (Krajcovicova‐Kudlackova et al., 2005). In a study conducted by Vangipurapu et al. (2019), they found that glycine and serine were associated with improved insulin sensitivity. Moreover, they were associated with significant increases in insulin secretion (DI), which may be responsible for lowered blood glucose. In addition, another study found that a plant‐based diet which leads to limited leucine and histidine can improve body composition and reduce insulin resistance (Kahleova et al., 2018).

Hruby and Jacques (2019) found that there was a significant inverse association between PP intake and Hs‐CRP. Moreover, they observed the same association between total protein intake and Hs‐CRP, which is also in line with the results of the present study. In a meta‐analysis conducted by Haghighatdoost et al. (2017), a significant inverse association between vegetarian diet and Hs‐CRP was found. Another study conducted on adults with chronic kidney disease showed an inverse association between consuming plant protein and levels of Hs‐CRP (Aycart et al., 2021). Various biological factors affect the levels of inflammatory factors, among which are the compounds found in plant sources. Bioactive peptides from plant sources have the ability to reduce inflammatory factors by regulating metabolic cycles that cause inflammation, such as the MAPK (mitogen‐activated protein kinase) pathway and NF‐Κb, which is the key pathway of protein phosphorylation (Liu et al., 2022).

## 5 CONCLUSIONS

In the present study among elders, it was found that higher intake of PP was more beneficial than AP, because it had more favorable associations with cardiovascular and inflammatory outcomes including WC, FBS, and Hs‐CRP compared with AP which was associated with lower blood pressure. However, it should be considered that the higher dairy product intake was responsible for the association.

## 6 LIMITATION AND STRENGTHS

To the best of our knowledge, this is the first study to investigate the relationship between dietary total, animal, and plant protein intake with cardiovascular outcomes and inflammatory factors in Iranian elderly men. It is worth noting several limitations such as a small sample size and the cross‐sectional design of the study. More comprehensive studies should be conducted. Moreover, it was not possible to make causal inferences because of the cross‐sectional design.

## AUTHOR CONTRIBUTIONS

**Hanieh Abbasi:** Conceptualization (equal); formal analysis (equal); investigation (equal); methodology (lead); software (equal); writing – original draft (lead); writing – review and editing (equal). **Noushin Fahimfar:** Formal analysis (equal); project administration (equal); software (equal); writing – review and editing (equal). **Pamela J. Surkan:** Writing – review and editing (supporting). **Leila Azadbakht:** Conceptualization (equal); data curation (lead); formal analysis (supporting); funding acquisition (lead); investigation (supporting); methodology (equal); project administration (lead); resources (equal); supervision (lead); validation (lead); visualization (lead); writing – original draft (supporting); writing – review and editing (supporting).

## CONFLICT OF INTEREST STATEMENT

The authors declare no conflicts of interest.

## Data Availability

The data that support the findings of this study are available on request from the corresponding author. The data are not publicly available due to privacy or ethical restrictions.
